# Editorial: Fecal Microbiota Transplants: challenges in translating microbiome research to clinical applications

**DOI:** 10.3389/fmicb.2025.1704947

**Published:** 2025-10-10

**Authors:** Emidio Scarpellini, Ludovico Abenavoli, Tetyana Falalyeyeva, Nazarii Kobyliak, Ghizlane Bendriss

**Affiliations:** ^1^Translational Research in GastroIntestinal Disorders (TARGID), Biomedical Sciences Group, KULeuven, Leuven, Belgium; ^2^Department of Health Sciences, University Magna Graecia of Catanzaro, Catanzaro, Italy; ^3^Department of Biomedicine, Taras Shevchenko National University of Kyiv, Kyiv, Ukraine; ^4^Department of Pathology, Medical Laboratory Care and Safe Diagnostics (CSD), Kyiv, Ukraine; ^5^Endocrinology Department, Bogomolets National Medical University, Kyiv, Ukraine; ^6^Premedical Department, Weill Cornell Medicine Qatar, Doha, Qatar

**Keywords:** fecal microbiota transfer (FMT), gut microbiome, precision medicine, clinical trial, artificial intelligence

Gut microbiota encompasses a complex ecosystem including bacteria, viruses, protozoa, yeasts, fungi, and archea (Seekatz, 2025). It has a multifaceted functional profile affecting human host metabolism, nutrient absorption, immune system education and tolerance and nervous system functioning, with impact on autism, depression (Li et al., 2025). With accumulating evidence on the extra-digestive communication between the gut and other organs via short-chain fatty acids, the vagus nerve, or cytokines, the gut microbiome has become a key therapeutic target (Bendriss et al., 2023).

This Research Topic aims at gathering multidisciplinary insights into the clinical application of fecal microbiota transplantation (FMT) across a broad range of conditions. Lately, FMT has been implemented to treat *Clostridium difficile* resistant infections, with excellent results, leading to its recognition by regulatory bodies as a life-saving therapy (Herman et al., 2025). Nevertheless, its broader use brings forward important questions regarding efficacy, safety, donor screening, and regulatory frameworks. With over 500 clinical trials currently investigating its potential in gastrointestinal, neurological, metabolic, and immune-mediated disorders, FMT is increasingly recognized as a central tool in the translation of microbiome research into practice. The contributions in this Research Topic explore key methodological, mechanistic, ethical, and clinical aspects of FMT—ranging from delivery strategies and treatment variability to host–microbiome interactions. Several studies also consider the role of computational tools, including artificial intelligence and metabolomic profiling, in enhancing predictability and safety. Together, these works reflect the growing need to standardize, refine, and ethically frame FMT as part of a precision medicine approach. Its modulation via prebiotics, probiotics, and postbiotics has seen several advancements in human disease over the last decades (Scarpellini et al., 2021). In detail, an increasingly personalized approach has taken place in research and current clinical practice (Baldi et al., 2025).

FMT has been explored in treatment of Crohn's disease, ulcerative colitis, irritable bowel syndrome, and neurological conditions such as Alzheimer's and Parkinson's disease, to improving response to cancer immunotherapy treatments (Al-Ali et al., 2021). However, there is a lack of objective markers of its efficacy across such a wide spectrum of research and clinical applications. In particular, it is crucial to measure the extent of engraftment following FMT to assess patients' response to treatment. More specifically, the gray zones in FMT investigation are community coalescence, which aims to study microbiome shifts following FMT engraftment; indicator features, which aim to assess specific microbiome features as a signal of engraftment; resilience, which aims to assess post-FMT recipients' microbiomes' resistance to shifts (Cymbal et al., 2025).

The contributions gathered in this Research Topic extend well beyond the established use of FMT for recurrent *Clostridioides difficile* infections and illustrate the breadth of its translational potential. Evidence now supports meaningful clinical benefits in functional and metabolic disorders: a systematic review demonstrated that FMT provides remission and symptom improvement in chronic constipation with favorable safety and microbial remodeling (Wang et al.), while preclinical work showed that gut microbial transfer can ameliorate hyperuricemia (Yuan et al.) and high-fat diet–induced obesity (Men et al.). Neurological and psychiatric conditions have also emerged as promising targets, with studies documenting improvements in chronic insomnia (Fang et al.), disease modification in Alzheimer's models (Xiang et al.), and integrative microbiome–metabolome signatures linked to attention-deficit/hyperactivity disorder (Lu et al.), alongside genetic evidence for causal associations between the gut microbiome and anorexia nervosa (Xia et al.).

In oncology, microbial modulation is increasingly recognized as a key determinant of therapeutic efficacy. FMT was found to suppress oncogenic programs in colorectal cancer (Han et al.) and to enhance the efficacy of 5-fluorouracil in pancreatic cancer (Li et al.) highlighting opportunities to optimize immuno- and chemotherapy responsiveness. Similarly, immune and autoimmune conditions are gaining attention: a comprehensive synthesis outlined the role of gut microbiota in shaping autoimmune disease progression and treatment strategies (Adawi), while experimental work demonstrated that FMT could alleviate lipopolysaccharide-induced osteoporosis by regulating microbial communities and lncRNA-TUG1 (Ma et al.). Microbiota transfer has also been shown to benefit chronic liver disease associated with hepatitis B virus infection, with improvements in both metabolic and microbial parameters (Deng et al.).

These advances also underscore the importance of donor factors and methodology. A study on healthy donors revealed that prior antibiotic exposure can durably alter microbial composition, phage dynamics, and resistance gene profiles, raising critical safety considerations (Karimianghadim et al.). At the same time, innovative delivery methods are being developed: an encapsulation protocol has demonstrated feasibility for stable, capsule-based administration (Sipos et al.). Expanding the scope even further, animal models illustrate how FMT influences systemic immune and neural processes, with demonstrated protection against pediatric traumatic brain injury (Fagan et al.) and mitigation of acute lung injury through anti-inflammatory and microbial pathways (Hua et al.).

Across this Research Topic, mechanistic insights converge on the role of transferable metabolic signatures—including SCFA production, bile acid pathways, and amino acid metabolism—in mediating host responses (Deng et al.; Lu et al.; Men et al.). Finally, forward-looking perspectives emphasize the urgent need for standardization and predictive tools: multi-omics integration and intelligence are proposed as key strategies to monitor engraftment, anticipate therapeutic outcomes, and tailor donor–recipient matching, bringing FMT closer to a precision medicine framework (Larsen and Brummer; Liu et al.; Cantón et al., 2024). Together, these studies demonstrate both the promise and complexity of FMT, reinforcing the need for careful methodological refinement and ethical oversight as the field moves toward clinical translation.

This Research Topic allows experts in the field to update their knowledge on the concept of gut microbiota, FMT, its use in clinical and research practice to beneficially affect human health.
